# Exploiting public databases of genomic variation to quantify evolutionary constraint on the branch point sequence in 30 plant and animal species

**DOI:** 10.1093/nar/gkad970

**Published:** 2023-11-11

**Authors:** Adéla Nosková, Chao Li, Xiaolong Wang, Alexander S Leonard, Hubert Pausch, Naveen Kumar Kadri

**Affiliations:** Animal Genomics, ETH Zürich, Universitätstrasse 2, 8092 Zürich, Switzerland; Animal Genomics, ETH Zürich, Universitätstrasse 2, 8092 Zürich, Switzerland; International Joint Agriculture Research Center for Animal Bio-Breeding, Ministry of Agriculture and Rural Affairs/Key Laboratory of Animal Genetics, Breeding and Reproduction of Shaanxi Province, College of Animal Science and Technology, Northwest A&F University, Yangling 712100, China; International Joint Agriculture Research Center for Animal Bio-Breeding, Ministry of Agriculture and Rural Affairs/Key Laboratory of Animal Genetics, Breeding and Reproduction of Shaanxi Province, College of Animal Science and Technology, Northwest A&F University, Yangling 712100, China; Animal Genomics, ETH Zürich, Universitätstrasse 2, 8092 Zürich, Switzerland; Animal Genomics, ETH Zürich, Universitätstrasse 2, 8092 Zürich, Switzerland; Animal Genomics, ETH Zürich, Universitätstrasse 2, 8092 Zürich, Switzerland

## Abstract

The branch point sequence is a degenerate intronic heptamer required for the assembly of the spliceosome during pre-mRNA splicing. Disruption of this motif may promote alternative splicing and eventually cause phenotype variation. Despite its functional relevance, the branch point sequence is not included in most genome annotations. Here, we predict branch point sequences in 30 plant and animal species and attempt to quantify their evolutionary constraints using public variant databases. We find an implausible variant distribution in the databases from 16 of 30 examined species. Comparative analysis of variants from whole-genome sequencing shows that variants submitted from exome sequencing or false positive variants are widespread in public databases and cause these irregularities. We then investigate evolutionary constraint with largely unbiased public variant databases in 14 species and find that the fourth and sixth position of the branch point sequence are more constrained than coding nucleotides. Our findings show that public variant databases should be scrutinized for possible biases before they qualify to analyze evolutionary constraint.

## Graphical Abstract

**Graphical Abstract:**
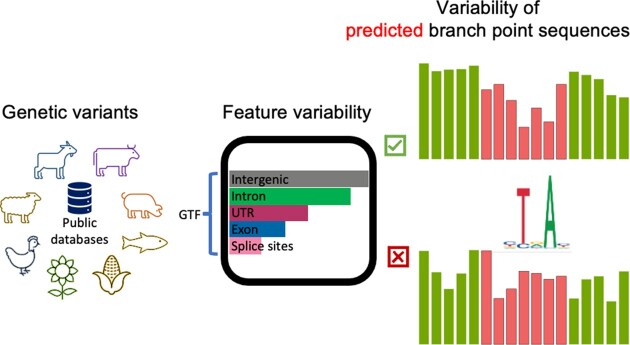

## Introduction

Precursor messenger RNA (pre-mRNA) splicing is executed by the spliceosome, a large ribonucleoprotein complex that assembles at the intron-exon boundary (1). Intronic features involved in the recognition and assembly of the spliceosome include the splice sites, polypyrimidine tract and branch point sequence (BPS). A degenerate heptamer containing the branch point residue constitutes the BPS (2). This motif usually resides within 50 bases upstream of the 3′ splice site in most of the introns across all eukaryotes. The heptamer includes highly conserved thymine and adenine residues at positions 4 and 6 respectively, with the adenine residue acting as branch point during pre-mRNA splicing (3–5).

Mutations overlapping the BPS can promote alternative splicing and manifest phenotype variation (6). However, despite their functional relevance, BPS are not readily accessible in most gene transfer files. Lack of experimentally proven branch points (7,8) and the degenerate nature of the sequence encompassing the branch point complicate systematic annotation of this regulatory motif.

Computational methods have been developed to predict BPS (4,9–11). The accuracy of these methods was assessed through benchmarking tests against experimentally proven BPs in humans and mice where BPP (9) and Branchpointer (11) emerged as the most accurate tools (12). Recently, Kadri *et al.* (13) predicted BPS in the human and bovine genomes and quantified their evolutionary constraint using exhaustive variant catalogues established from whole-genome sequencing (WGS). Their analyses showed that the BPS encompasses evolutionarily conserved thymine and adenine residues that are more strongly depleted for variants than coding sequences, suggesting that they are under extreme purifying selection. Recovery of strong signatures of constraint for nucleotides within predicted BPS also shows that the degenerate motif can be localized *in silico* with high accuracy. Variants affecting the evolutionarily conserved residues can have profound phenotypic consequences (9,13). Recent analyses in human genomes suggest that the fourth nucleotide of the heptamer might be more strongly depleted for variants than the branch point itself (14,15). However, it remains an open question if this constraint pattern is consistent across evolutionarily distant species.

Here we predict BPS in 30 plant and animal species and attempt to study their constraint using public variant databases. We uncover implausible variant distributions in 16 out of 30 public databases precluding such a study in all species. Investigation of variability of the BPS using unbiased public databases of genomic variation reveals strong evolutionary constraint on both the branch point and on the position two base pairs upstream in 14 species investigated.

## Materials and methods

### Whole-genome sequence variant databases

We used whole-genome sequencing (WGS) data of 139 pigs that were sequenced at an average read depth >8×. Sequence reads were processed and aligned against the Sscrofa11.1 reference sequence as detailed in (16). We called variants with DeepVariant (version 1.3.0, (17,18)), producing a gVCF file per sample. The gVCF files were then merged and filtered using GLnexus (version 1.4.1, Lin, MF., Rodeh O., Penn, J., Bai, X., Reid, JG., Krasheninina, O. and Salerno, WJ. (2018) GLnexus: joint variant calling for large cohort sequencing. biorXiv doi: 10.1101/343970, 11 June 2018, pre-print: not peer reviewed) with the DeepVariantWGS configuration, followed by imputation with Beagle 4.1 (19). Sequence variants were called previously for 266 cattle, 161 sheep and 157 goats (Table 1). We considered only biallelic sequence variants for our analyses.

**Table 1.** Datasets used for the constraint analyses

Species	*N*	SNPs (Ti/Tv)	% Coding (Ti/Tv)	Predicted branch points	Average density of variants (per 100 bp)	Reference genome	Source
Cow	266	29227950 (2.21)	0.91 (3.00)	179476	1.18	ARS-UCD1.2	(13)
Cow	-	89118442 (1.09)	3.27 (0.55)	179476	3.58	ARS-UCD1.2	EVA rs4
Cow (filtered)	-	34551781 (2.20)	0.49 (3.33)	179476	1.39	ARS-UCD1.2	EVA rs4
Cow (all COFACTOR)	-	34580719 (0.55)	7.35 (0.45)	179476	1.39	ARS-UCD1.2	EVA rs4
Pig	139	24074441 (2.36)	0.73 (3.28)	192744	1.04	Sscrofa11.1	This study
Pig	-	56883886 (1.96)	0.91 (2.38)	192744	2.47	Sscrofa11.1	Ensembl variation
Sheep	161	16198123 (2.59)	0.54 (4.35)	168025	0.60	Oar_rambouillet_v1.0	Li 2023, submitted
Sheep	-	48541784 (2.45)	0.82 (3.53)	168025	1.80	Oar_rambouillet_v1.0	Ensembl variation
Goat	157	13364058 (2.48)	0.66 (3.95)	174407	0.53	ARS1	(26)
Goat	-	31517363 (2.40)	0.68 (3.88)	174407	1.26	ARS1	Ensembl variation

### Public variant databases

We downloaded reference sequences and their annotations including non-coding RNAs, as well as a VCF file with polymorphic positions for 30 species from EVA (release 4, (20)), Ensembl (release 107, (21)) or Ensembl Plants (release 55, (22)). Access information for all data is provided in Supplementary Table S2.

We used these data to evaluate the number of variants, proportion of variants in protein-coding regions, average genome-wide variability (in variants per 100 bases) and transition to transversion (Ti/Tv) ratio. Variants overlapping exons, start codons and stop codons were considered as coding variants.

### Prediction of branch point sequences

We followed the approach of Kadri *et al.* (13) to predict BPS in 30 species using BPP (9). In short, we obtained coordinates of introns in protein-coding genes from GTF files of each species, mindful of gene-strand orientation. We used species-specific weighted octanucleotide frequencies estimated as suggested by Zhang *et al.* (9) and the position weight matrix of predicted human BPS for model training (9). For the analysis of constraint, we only considered the most probable BPS within each intron.

The variability between positions 4 and 6 of the heptamer was compared for each species with Fisher's exact test. We applied Bonferroni-correction to account for multiple testing (number of species tested).

### Variation in genic features

Variability was calculated as the number of variants per 100 bp divided by the respective species’ genome-wide variability for variants overlapping nine annotated genomic features (3′ and 5′ splice sites, start and stop codons, 3′ and 5′ UTR, introns, exons, intergenic regions) and predicted BPS. Genome-wide variability, i.e. average number of variants per 100 bp, was calculated as total number of variants divided by the size of the genome. The genome size was the total length of all chromosomes considered but undetermined bases (‘N’) were excluded.

### Genomic variant database analysis

Based on the analyses of WGS datasets we established three criteria to assess the quality of public databases. The criteria were (i) genome-wide variability of minimum 1 variable site per 1000 bp; (ii) variability in intergenic regions above the average genome-wide; (iii) depletion of variation at the 4 bases overlapping splice sites. Databases not fulfilling all criteria were excluded from further analyses (Supplementary Table S1, File S1). Eleven species that satisfied these criteria were considered to estimate constraint patterns.

## Results

Purifying selection against deleterious mutations manifests as a depletion of variation overlapping constrained nucleotides. We (13) and others (23,24) showed that counting the number of variable sites within functional classes of annotations enables quantifying constraints at nucleotide resolution. We hypothesized that this approach is applicable to validate and quantify evolutionary constraint on the branch point sequence (BPS) for any species for which an annotated reference genome and a large and unbiased variant database are available.

### Bovine public variant database is biased

**Figure 1.**
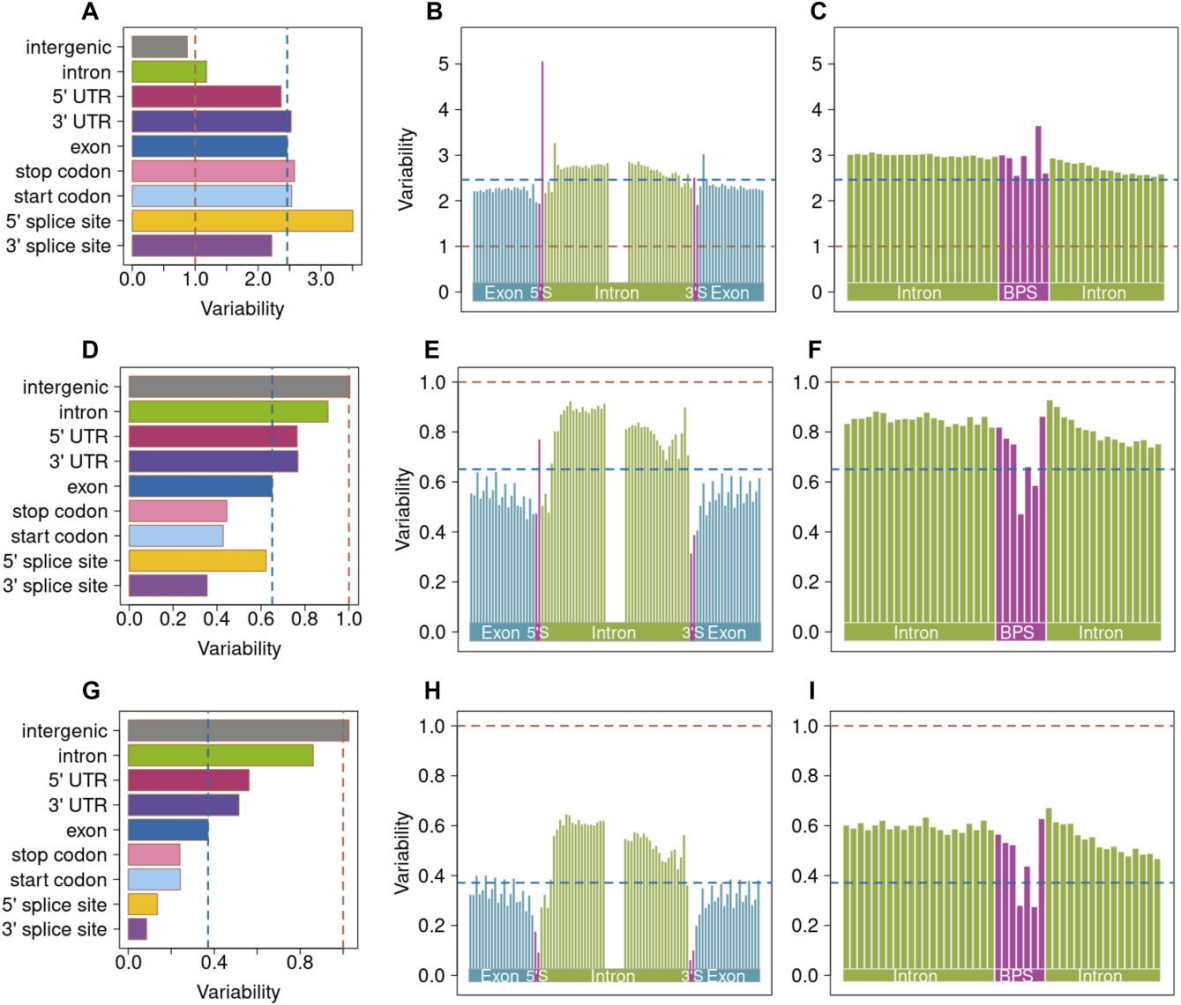
Variation in genomic features quantified using raw and filtered public bovine variant database. Variability of nine bovine genomic features (**A, D, G**), as well as nucleotide-wise constraint in and around the splice-sites (**B, E, H**) and predicted branch point sequences (**C, F, I**) using raw (top panels) and filtered (middle and bottom panels) variant databases. Constraint was quantified relative to average genome-wide variability using all 89 118 442 SNPs (top panels), a subset of 57 875 698 SNPs that did not contain variants only submitted by the COFACTOR_GENOMICS_CFG20140112 project (middle panels), and a subset of 34 551 781 SNPs that contained only SNPs that were submitted at least twice (bottom panels). Red and blue lines denote average genome-wide and exome variability, respectively.

We conducted a proof-of-concept study with 89 118 442 biallelic SNPs from the bovine EVA database (release 4; (20)) to investigate evolutionary constraint on BPS in the bovine genome. We calculated nucleotide-wise constraint—hereafter referred to as ’variability’—for each position of the BPS relative to the average genome-wide density of variants per 100 bp. Contrary to findings in a catalogue of variants established through WGS (13), the branch point was the least constrained nucleotide in the heptamer when variants from the public database were used (Figure 1C). Implausible constraint patterns were also evident for other well annotated features of the genome. For instance, we found intergenic regions to be less variable than coding regions (Figure 1A), and excessive variability at the splice sites (Figure 1B). These findings suggested that the bovine EVA database contains biased or erroneous variants.

A more than threefold higher proportion of coding variants in the EVA database than the WGS variant catalogue (3.27% versus 0.91%, Table 1) suggested that the EVA database contains variants discovered from exome sequencing. While the expected transition to transversion ratio (Ti/Tv) in coding regions is approximately 3 (25), it was implausibly low (0.55) in the EVA database, corroborating its contamination with erroneous variants. These irregularities were mainly due to a large batch of variants (*n* = 38 008 641) from one submitter that included many (7.45%) coding variants with very low Ti/Tv ratio (0.45). Most of these variants (83%; Supplementary Figure S1) were unvalidated, i.e. they were not confirmed by another submission. Once all variants private to this batch were removed, a subset of 57 875 698 EVA SNPs largely recovered the expected variability of the investigated features (Figure 1D–F). However, high variability of nucleotides overlapping the 5′ splice site and a relatively low Ti/Tv ratio (1.69) suggested that this subset is still biased. We repeated the analyses with 3 455 1781 variants that were submitted to EVA at least twice. Variants within this subset had a Ti/Tv ratio of 2.20. These variants recovered a pattern of evolutionary constraint on the nine genomic features and nucleotide-wise constraints on splice sites and branch point sequences that matched previous findings from WGS-derived variants (Figure 1G–I; Table 1). However, this subset contained fewer coding variants (0.49%) than the WGS-derived catalogue which suggests that strict filtration removed true rare coding variants from the data.

### Public variant databases reveal expected constraints in pig, sheep, and goat

We quantified constraint patterns in the same genomic features of three additional species to investigate if other public variant databases suffer from similar biases. These analyses were performed in pig, sheep, and goat for which exhaustive variant catalogues were available from both WGS and public databases (Table 1). The predicted BPS in 192 744 pig, 168 025 sheep and 174 407 goat introns had a ‘nnyTrAy’ consensus sequence that contained two conserved nucleotides at the branch point itself (position 6) and two bp upstream (position 4) (Supplementary Figure S2A). The branch points were at a median distance of 26 bp upstream of the 3′ splice site (Supplementary Figure S2B). Most BPS had a canonical ‘TnA’ motif at position 4–6 (88, 90 and 89% in pigs, sheep, and goats, respectively).

**Figure 2.**
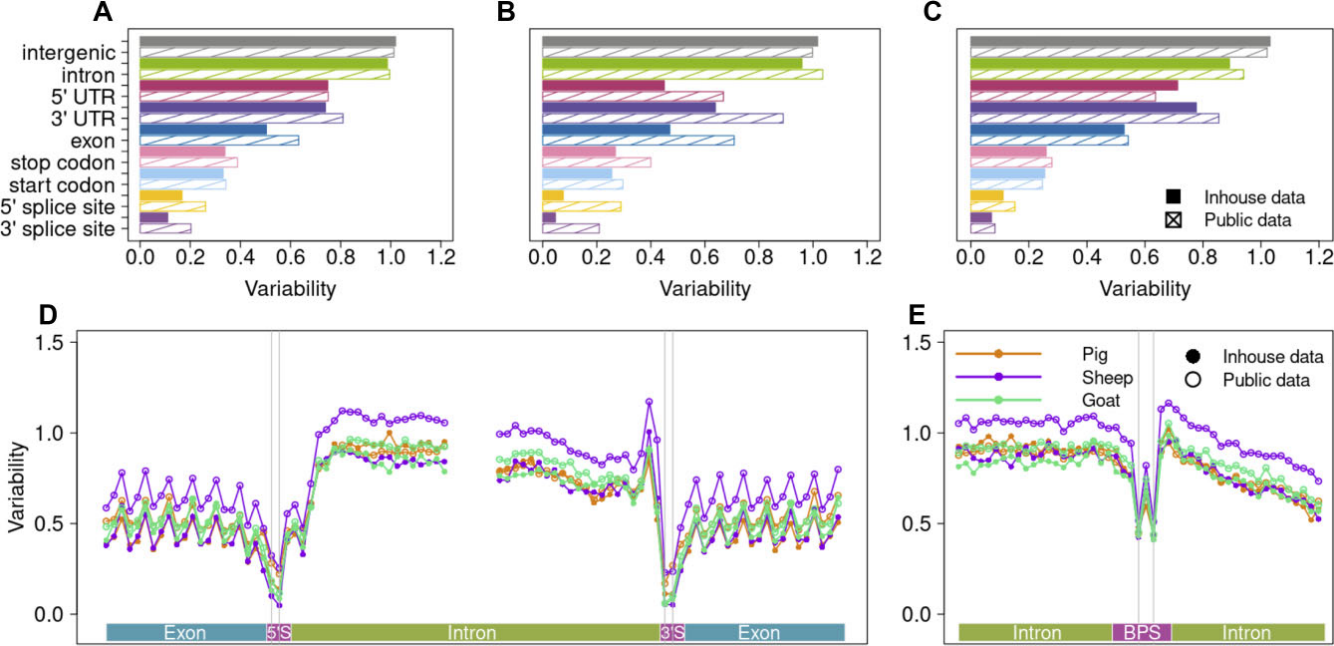
Variation in pig, sheep, and goat genomic features quantified through variants from whole-genome sequencing and public databases. Variability of the nine features of the pig (**A**), sheep (**B**) and goat genomes (**C**). Nucleotide-wise variation relative to average genome-wide variability in and around splice sites (**D**) and branch point sequence (**E**).

Variant discovery in WGS data of 139 pigs, 161 sheep and 157 goats yielded 24 074 441, 16 198 123 and 13 364 058 biallelic SNPs with Ti/Tv ratios between 2.36 and 2.59 (Table 1). Variability of the nine genomic features differed as expected and confirmed previously established patterns of constraint (Figure 2A–D). We also observed a striking depletion of variation on positions 4 and 6 of the predicted BPS (Figure 2E). The constraint on both nucleotides was stronger than on coding sequences but did not differ significantly between them (Fisher's exact test *P*-values 0.06, 0.79, 0.50 for pig, sheep and goat, respectively).

The public pig, sheep, and goat databases contained more than twice the number of variants we established through WGS but between 28% and 36% overlapped between the databases and WGS for the respective species (Table 1). Because the public databases aggregate variant information from many individuals from multiple breeds, the Ti/Tv ratios, proportion of coding variants, and constraints in functional features differed from those established with the smaller WGS subset but were within plausible ranges (Table 1; Figure 2A–D). Nucleotide-wise constraint in the BPS was also consistent with the pattern obtained from variants established through WGS (Figure 2E). As observed with variants from WGS, the constraint did not differ between positions 4 and 6 (Fisher's exact test *P*-values 0.48, 0.67, 0.47 for pig, sheep and goat, respectively).

### Variant bias is widespread in public databases

Exhaustive variant information from four public databases recovered evolutionary constraints similar to those established from WGS. This encouraged us to conduct a comparative analysis of constraint on the BPS in 26 additional species (18 animals from 13 orders and 8 plants from 4 orders) for which at least one million SNPs were available through EVA (*n* = 8) or Ensembl (*n* = 18) databases. We evaluate the quality of these databases prior to the comparative constraint analyses to ensure they are free from erroneous and biased variants.

Two public databases were excluded prior to the comparative analysis because variant density was too low (<1 variants/1000 bp). Variants from 13 databases were incongruent with properties of genome-wide variants and thus were not suitable for an unbiased comparative assessment of evolutionary constraint across species (Supplementary Table S1, File S1). The variability in intergenic regions was lower than the average genome-wide variability in 12 excluded databases possibly indicating biased variant distribution due to exome sequencing. An excess of exonic variants in five of these databases is further evidence that the variants fail to represent genome-wide variability. The well-established constraint at the four positions overlapping the 3′ and 5′ splice sites was absent in five databases (File S1).

Other variant characteristics such as the proportion of coding variants or the Ti/Tv ratio were not abnormal for many excluded databases, indicating that these parameters are not suitable to assess the plausibility of variant databases. For instance, a Ti/Tv ratio of 1.96 and 1.11% coding variants in the *Equus caballus* database are compatible with expectations for genome-wide variants (Supplementary Table S1). Yet, variants from this database revealed an implausibly high variability at both splice sites (three times the genome wide variability at 5′ SS and two times at 3′ SS) and downstream the BPS at intronic positions overlapping the polypyrimidine tract (File S1). A plausible Ti/Tv ratio (1.87) and percentage of coding variants (2.89%) may suggest that the *Gallus gallus* variant database is representative for whole-genome variability in chicken. However, an excess of variability of the nucleotides overlapping the 5′ splice sites is implausible. Variants from this database also uncovered a constraint pattern in the BPS which deviates from what we established in unbiased variant catalogues.

**Figure 3.**
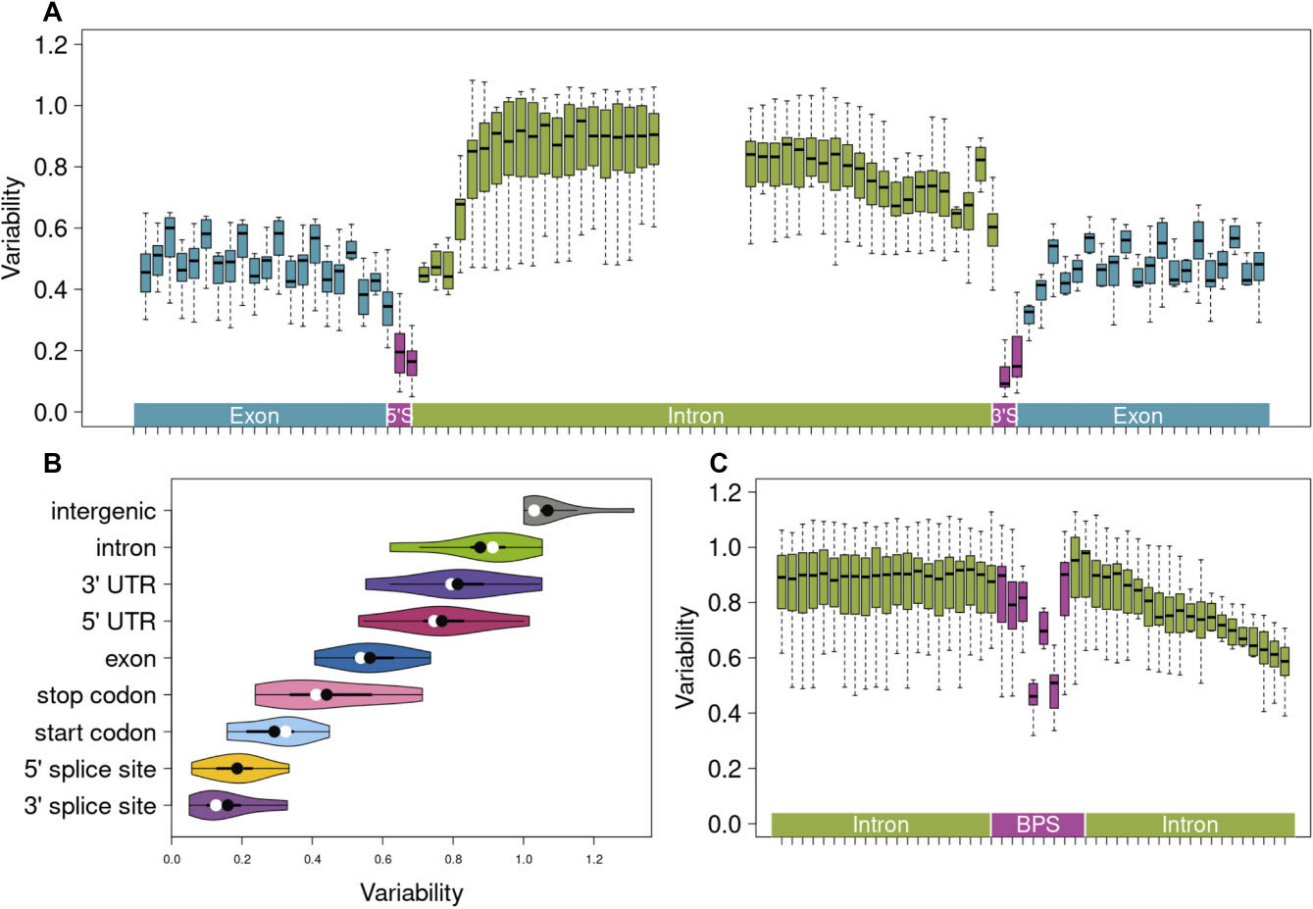
Variation in genomic features across 11 species quantified using public variant databases. Boxplots of nucleotide-wise variability relative to average genome-wide variability in and around splice sites (**A**) and predicted branch point sequences (**C**). Violin plots of variability in nine genomic features. Means and medians are indicated with black and white circles, respectively (**B**).

Only 11 public variant databases (File S1, Supplementary Table S1) fulfilled our criteria, i.e. they contained on average at least one variant per 1000 bp, variant density was higher in intergenic regions than genome-wide (Figure 3B) and constraint on the 3′ and 5′ splice sites were evident (Figure 3A). These databases contained between 4 486 640 and 69 472 724 variants of which between 0.61% and 16.51% overlapped coding sequences. We subsequently conducted a comparative analysis of BPS variation in these species.

A vast majority of the predicted BPS for these 11 species contained the canonical ‘TnA’ motif overlapping positions 4–6 of the heptamer (between 90% in *Pan troglodytes* and 98% in *Phaseolus vulgaris*; Supplementary Table S1, Supplementary Figure S3). The predicted branch points were primarily between 14 and 145 bp upstream of the 3′ splice site with median distance of 27 bp (Supplementary Figure S4), consistent with BPS placement in other species. The comparative analysis of BPS variation in these 11 species revealed strong constraint on positions 4 and 6 (Figure 3C, Supplementary Table S1). The constraint between these two positions differed significantly in four of the 11 species investigated (Bonferroni corrected Fisher's exact *P* < 4.5 × 10^−3^). In three of those four species, the constraint was stronger on the position 4 than on the position 6 (Supplementary Table S1).

## Discussion

Our comparative analysis of branch point sequence (BPS) variation relied on computationally predicted BPS because exhaustive catalogues of experimentally proven branch points were not available for the species considered. While the length of the reported consensus sequence encompassing the branch point varies from five bases in humans (27) to ten bases in plants (5), all BPS in our study were heptamers that contained the branch point at the sixth position. Constraints on positions 4 and 6 were striking in all species investigated, corroborating a pivotal role of both residues for spliceosome assembly during pre-mRNA splicing (7). However, we did not find conclusive evidence for higher constraint on position 4 of the heptamer across the 14 plant and animal species considered, as reported for human BPS (14,15). The set of computationally predicted BPS may contain errors and inaccuracies that have prevented us from distinguishing constraints between two similarly constrained nucleotides. Further research involving well-curated variant catalogues and experimentally proven branch points is needed to investigate if the pattern of constraint for these two nucleotides is consistent across species. Our findings confirm that an adenine residue is the most preferred branch point, and that thymine is the most preferred residue at 2 bp upstream of the branch point (28). High evolutionary conservation of these nucleotides across distant eukaryotic species reiterates the need to consider them in search for trait-associated variants.

We use public variant databases to assess evolutionary constraint on different genomic features. (29–31) Variants for the 30 species considered are accessible from three widely used public databases, but our approach can be extended to any other variant catalogue (32–34). Contamination of some databases with erroneous or biased variants caused implausible constraint patterns. Thus, our study corroborates that variants from public databases need to be evaluated carefully due to their partly unknown origin and lack of curation (29,31,35–37). Strict filtration, such as the removal of variants that were submitted only once, was required to recover expected constraint patterns from a public bovine variant database. This approach is only possible with accompanying metadata, which is not always available. Moreover, this approach also removes true rare variants enriched in evolutionary constraint signatures, and as such is not generally advisable. While we demonstrate the usefulness of public databases, we advocate rigorous curation and mandatory inclusion of metadata with each submission to ensure appropriate use of these valuable resources.

Assessment of constraint patterns in well-annotated genomic features is more useful to evaluate the quality of variant databases than inspecting other widely used parameters such as Ti/Tv ratio. We show that the constraint on the splice sites and the proportion of variants in intergenic regions are the most informative for such an assessment. By using a simple and straightforward approach of counting variable sites overlapping genomic features, we show that erroneous and biased variants contaminate 16 of the 30 investigated public variant databases. Since these constraint patterns are so widespread, such an approach may provide quality assessment for existing or even purely predictive annotations.

## Supplementary Material

gkad970_Supplemental_Files

## Data Availability

The data underlying this article are available in the European Nucleotide Archive (ENA) at http://www.ebi.ac.uk/ena, and can be accessed under accession codes PRJEB38156, PRJEB37956 and PRJEB39374. The funding bodies were neither involved in the design of the study and collection, analysis, and interpretation of data nor in writing the manuscript.
