# Supplementary material for: Exploiting public databases of genomic variation to quantify evolutionary constraint on the branch point sequence in 30 plant and animal species
Source: Nucleic Acids Res. 2023 Nov 11;51(22):12069–75. doi: 10.1093/nar/gkad970 (PMC10711541; doi:10.1093/nar/gkad970)
Supplement: gkad970_Supplemental_Files [file gkad970_supplemental_files.zip › Supplementary_Figures.pdf]

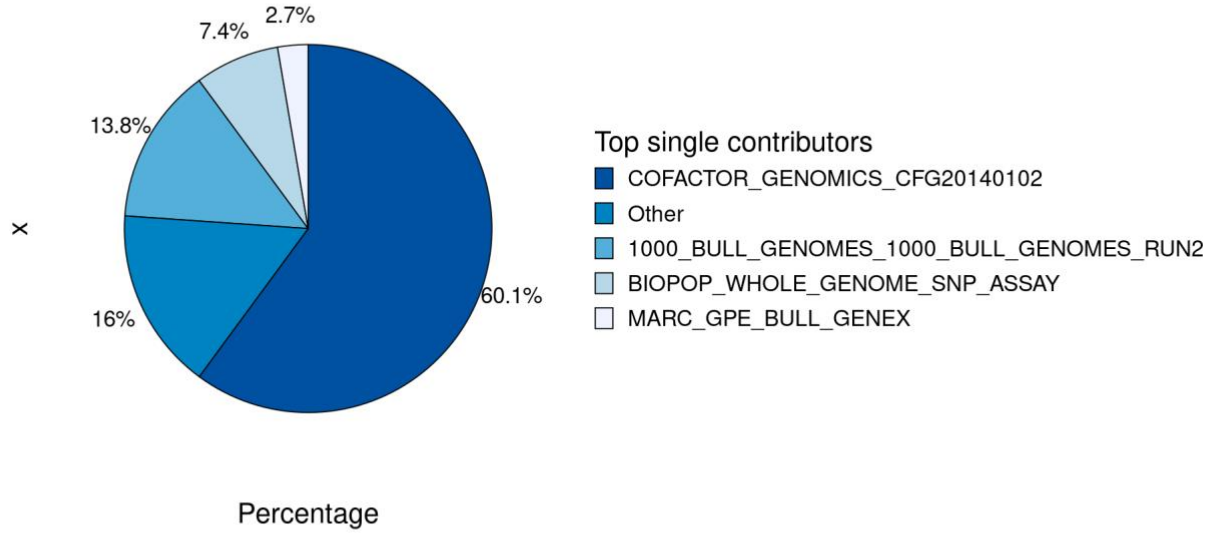

Figure S1: **Main contributors of single-entry variants.** Top four contributors submitted 84 % of all (N = 51,359,349) singletons. The largest batch (N = 31,580,941) of unvalidated variants was submitted by COFACTOR_GENOMICS_CFG20140112.

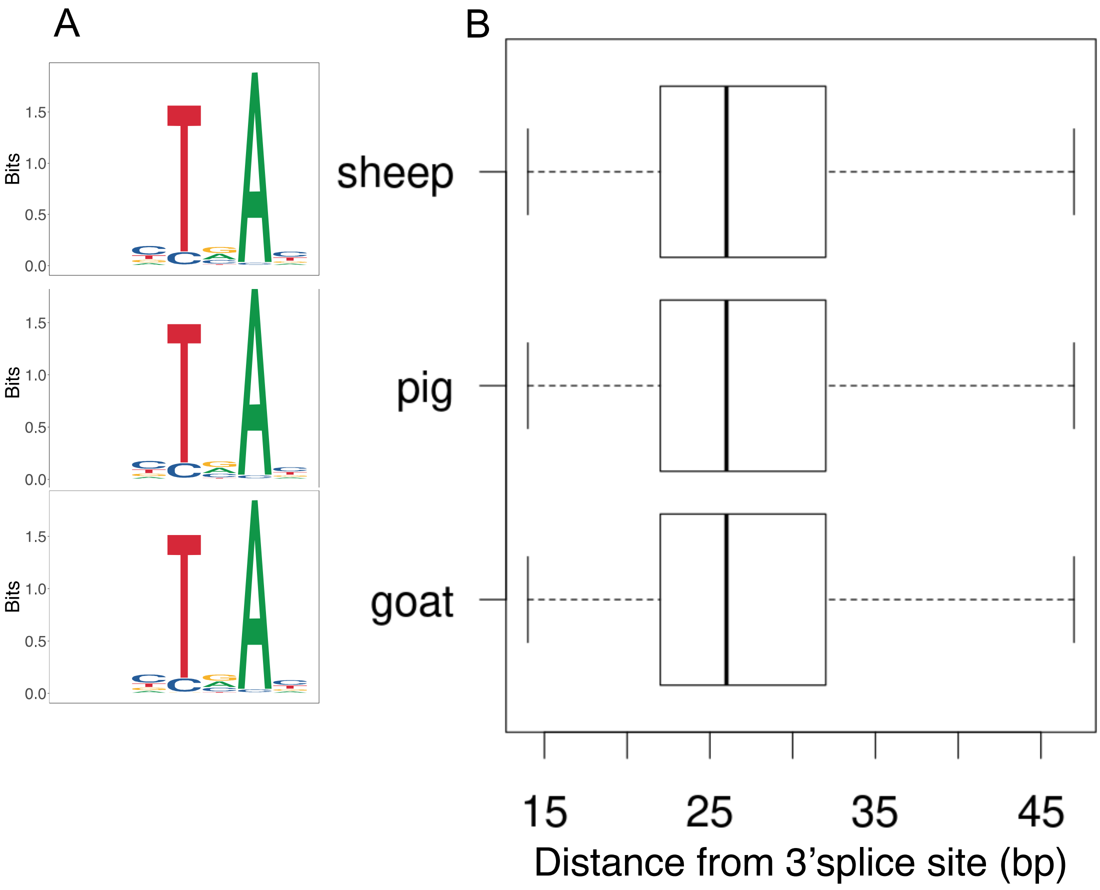

Figure S2: Predicted branch point sequences in sheep, pig and goat. (A) Motif logos of predicted branch point consensus sequences and (B) their placement (distance from 3' splice site in base pairs) in the sheep, pig and goat genomes.

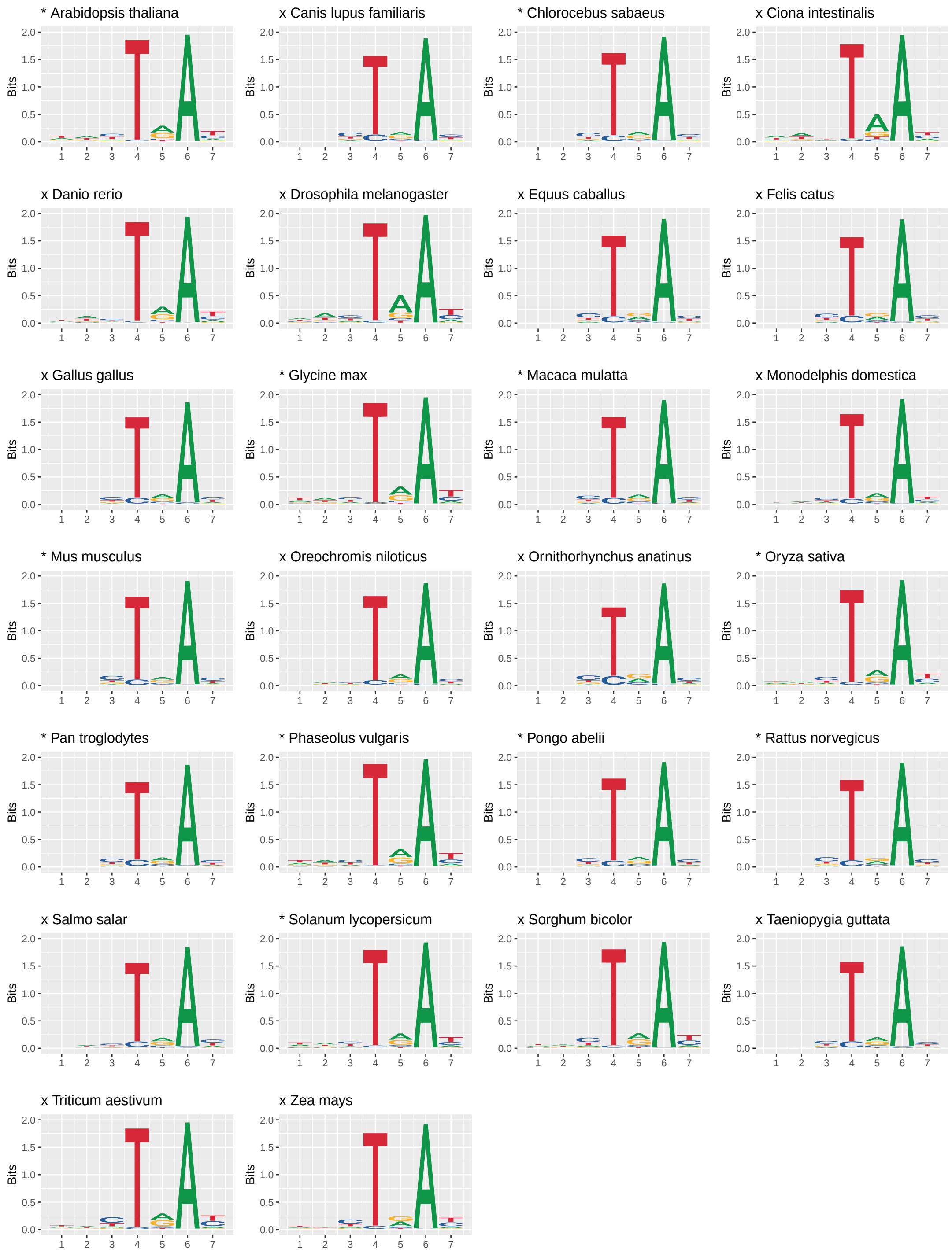

Figure S3: **Predicted branch point consensus sequences in 26 species.** Variant databases for Eleven species (marked with asterisk) passed our criteria, while 15 species (marked with ‘X’) failed for following reasons: two species had too low genome-wide variability (*Monodelphis domestica, Ornithorhynchus anatinus)*, 12 species had intergenic variability lower than the genome-wide (*Canis lupus familiaris, Ciona intestinalis, Danio rerio, Drosophila melanogaster, Felis catus, Gallus gallus, Oreochromis niloticus, Ornithorhynchus anatinus, Salmo salar, Taeniopygia guttata, Triticum aestivum, Zea mays*), and five species revealed implausible constraint at the splice sites (*Equus caballus, Gallus gallus, Salmo salar, Sorghum bicolor and Triticum aestivum*).

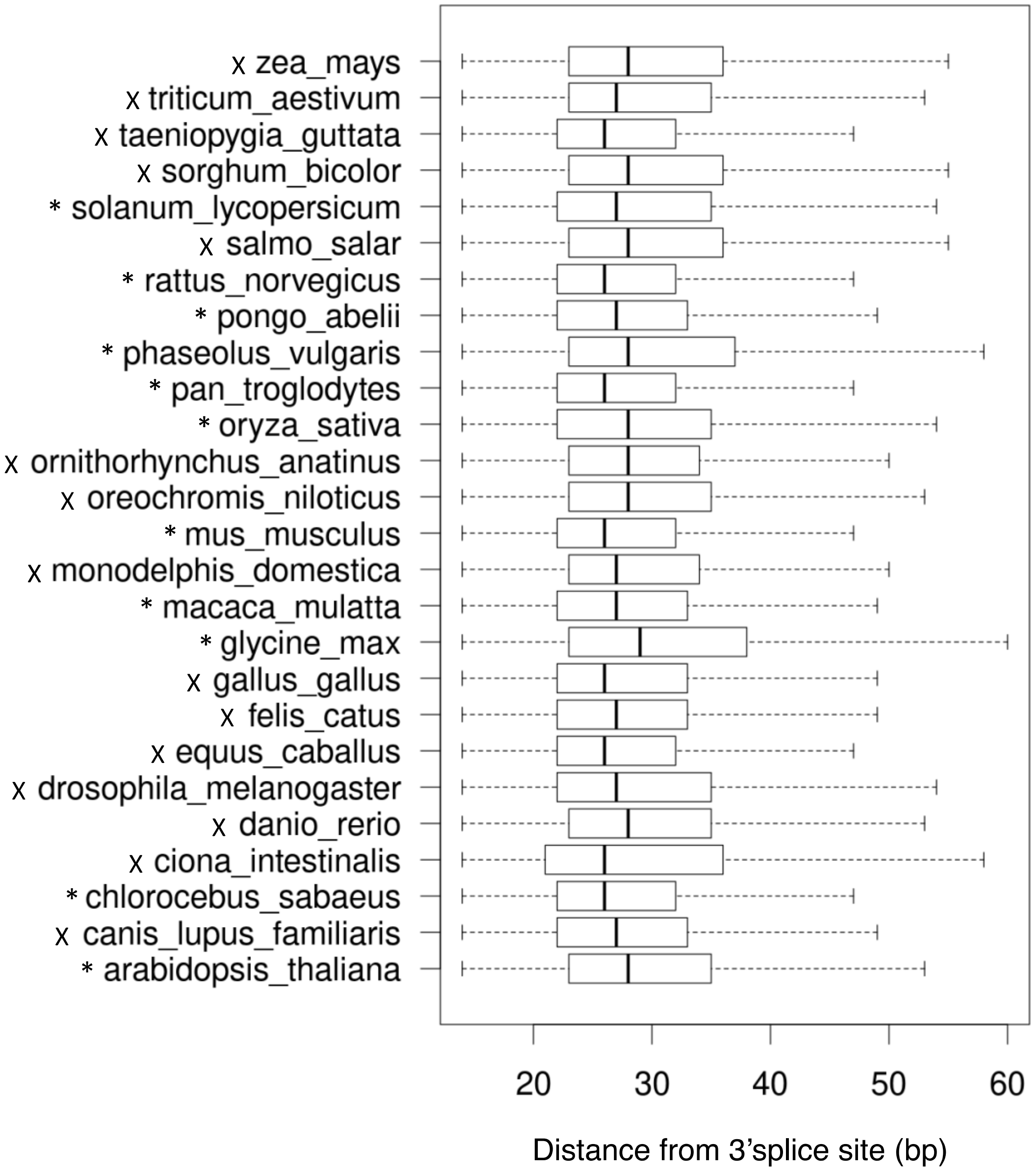

Figure S4: Placement of predicted branch points in 26 species. Variant databases for Eleven species (marked with asterisk) passed our criteria, while 15 species (marked with 'X') failed for following reasons: two species had too low genome-wide variability (*Monodelphis domestica, Ornithorhynchus anatinus)*, 12 species had intergenic variability lower than the genome-wide (*Canis lupus familiaris, Ciona intestinalis, Danio rerio, Drosophila melanogaster, Felis catus, Gallus gallus, Oreochromis niloticus, Ornithorhynchus anatinus, Salmo salar, Taeniopygia guttata, Triticum aestivum, Zea mays*), and five species revealed
